# Supplementary material for: A phylogenetically-conserved axis of thalamocortical connectivity in the human brain
Source: Nat Commun. 2023 Sep 27;14:6032. doi: 10.1038/s41467-023-41722-8 (PMC10533558; doi:10.1038/s41467-023-41722-8)
Supplement: Supplementary file 3 — Description of Additional Supplementary Files [file 41467_2023_41722_MOESM3_ESM.pdf]

Description of Additional Supplementary Files:

Supplementary Data 1: The concatenated connectivity and gene expression matrix for all 921 thalamic seeds across 250 cortical regions and 2228 genes for the human data.

Supplementary Data 2: The first 500 components of the PCA on the concatenated connectivity and gene expression human data.

Supplementary Data 3: The concatenated connectivity and gene expression matrix for all 35 thalamic nuclei across 38 cortical regions and 447 genes for the mouse data.

Supplementary Data 4: The PCA results for the concatenated connectivity and gene expression mouse data.

Supplementary Data 5: Loadings for human and mouse genes across PC1, PC2, and PC3. Also lists overlapping genes between human and mouse datasets.

Supplementary Data 6: A list of the 100 genes with the highest/lowest loadings for PC1, PC2, and PC3.

Supplementary Data 7: Enrichment for cell class, neuron subtype, and neuron subcluster for the top 100 genes with the highest/lowest loadings for PC1.

Supplementary Data 8: Enrichment for cell class, neuron subtype, and neuron subcluster for the top 100 genes with the highest/lowest loadings for PC2.

Supplementary Data 9: Enrichment for cell class, neuron subtype, and neuron subcluster for the top 100 genes with the highest/lowest loadings for PC3.

Supplementary Data 10: List of genes showing significant differential expression across development for PC1, PC2, and PC3.

Supplementary Data 11: Enrichment for disease for the top 100 genes with the highest/lowest loadings for PC1. Also lists the genes linked to significantly enriched diseases.

Supplementary Data 12: Enrichment for disease for the top 100 genes with the highest/lowest loadings for PC2. Also lists the genes linked to significantly enriched diseases.

Supplementary Data 13: Enrichment for disease for the top 100 genes with the highest/lowest loadings for PC3. Also lists the genes linked to significantly enriched diseases.
