# Supplementary material for: A phylogenetically-conserved axis of thalamocortical connectivity in the human brain
Source: Nat Commun. 2023 Sep 27;14:6032. doi: 10.1038/s41467-023-41722-8 (PMC10533558; doi:10.1038/s41467-023-41722-8)
Supplement: Supplementary file 17 — Reporting Summary [file 41467_2023_41722_MOESM17_ESM.pdf]

nature portfolio

Corresponding author(s): Stuart Oldham

Last updated by author(s): 26/8/23

Reporting Summary

Nature Portfolio wishes to improve the reproducibility of the work that we publish. This form provides structure for consistency and transparency in reporting. For further information on Nature Portfolio policies, see our Editorial Policies and the Editorial Policy Checklist.

Statistics

For all statistical analyses, confirm that the following items are present in the figure legend, table legend, main text, or Methods section.

n/a	Confirmed	
☐	☒	The exact sample size (n) for each experimental group/condition, given as a discrete number and unit of measurement
☐	☒	A statement on whether measurements were taken from distinct samples or whether the same sample was measured repeatedly
☐	☒	The statistical test(s) used AND whether they are one- or two-sided<br>*Only common tests should be described solely by name; describe more complex techniques in the Methods section.*
☐	☒	A description of all covariates tested
☐	☒	A description of any assumptions or corrections, such as tests of normality and adjustment for multiple comparisons
☐	☒	A full description of the statistical parameters including central tendency (e.g. means) or other basic estimates (e.g. regression coefficient) AND variation (e.g. standard deviation) or associated estimates of uncertainty (e.g. confidence intervals)
☐	☒	For null hypothesis testing, the test statistic (e.g. F, t, r) with confidence intervals, effect sizes, degrees of freedom and P value noted<br>*Give P values as exact values whenever suitable.*
☒	☐	For Bayesian analysis, information on the choice of priors and Markov chain Monte Carlo settings
☒	☐	For hierarchical and complex designs, identification of the appropriate level for tests and full reporting of outcomes
☐	☒	Estimates of effect sizes (e.g. Cohen's d, Pearson's r), indicating how they were calculated

Our web collection on statistics for biologists contains articles on many of the points above.

Software and code

Policy information about availability of computer code

Data collection	Open datasets were used. No primary data was collected for this study.
Data analysis	Code used for data processing, analysis, and figure generation directly relies on the following software:<br>Python (v3.8.5); BrainSMASH (v0.11.0), neuromaps (v0.0.3), scipy (v1.5.2), pandas (v1.1.3), numpy (v1.19.2), parcellation_fragmenter (https://github.com/miykael/parcellation_fragmenter), nibabel (v3.0)<br>R (3.6.0); nlme (v3.1-161), mgcv (v1.8-41), tidyverse (v1.3.2), DescTools (v 0.99.47)<br>Connectome Workbench (v1.5.0)<br>MATLAB (r2020a)<br>FreeSurfer (v5.3.0)<br>FSL (v5.0.11)<br>MRtrix3 (v3.0.15)<br>WEB-based GEne SeT AnaLysis Toolkit (v2019)<br><br>Custom code: https://github.com/StuartJO/ThalamicGradients

For manuscripts utilizing custom algorithms or software that are central to the research but not yet described in published literature, software must be made available to editors and reviewers. We strongly encourage code deposition in a community repository (e.g. GitHub). See the Nature Portfolio guidelines for submitting code & software for further information.

Data

Policy information about availability of data

All manuscripts must include a data availability statement. This statement should provide the following information, where applicable:

- Accession codes, unique identifiers, or web links for publicly available datasets
- A description of any restrictions on data availability
- For clinical datasets or third party data, please ensure that the statement adheres to our policy

The following open access datasets were used:

Human Connectome Project (HCP): Diffusion and structural MRI data for 100 adults from the S1200 subjects data release; https://www.humanconnectome.org/study/hcp-young-adult/data-releases

Allen Human Brain Atlas, voxelwise smoothed expression maps: Voxelwise expression maps for 2233 genes; http://www.meduniwien.ac.at/neuroimaging/mRNA.html

List of the top 500 differentially expressed genes across the mouse thalamus and their associated PC1 scores: https://static-content.springer.com/esm/art%3A10.1038%2Fs41593-019-0483-3/MediaObjects/41593_2019_483_MOESM3_ESM.xlsx

List of 2,413 genes showing elevated expression in the human brain: https://static-content.springer.com/esm/art%3A10.1038%2Fs41593-018-0195-0/MediaObjects/41593_2018_195_MOESM4_ESM.xlsx

Allen Mouse Brain Atlas: Gene expression data for 19419 genes across 213 regions in the mouse brain; https://doi.org/10.5281/zenodo.4609603

Allen Mouse Connectivity Atlas: Axonal tracing data for 213 regions in the mouse brain; https://doi.org/10.5281/zenodo.4609603

Allen Mouse Brain CCFv3: Atlas NiFti volume and flat map for visualising mouse data; https://scalablebrainatlas.incf.org/mouse/ABA_v3#downloads, http://download.alleninstitute.org/publications/allen_mouse_brain_common_coordinate_framework/cortical_surface_views/ccf/annotation/

Mouse cortical properties: List of nine measures of cortical organisation in the mouse; https://doi.org/10.6084/m9.figshare.7775684.v1

Mouse brain hierarchies: Measure of mouse brain hierarchy (based on cortico-cortical, thalamo-cortical, and cortico-thalamic connections); https://github.com/AllenInstitute/MouseBrainHierarchy/

DropViz: Drop-seq analysis of 89,027 cells in the adult mouse thalamus; http://dropviz.org

The Human Protein Atlas: List of genes with protein expression in the human thalamus; https://v21.proteinatlas.org/humanproteome/brain/thalamus

Ensembl Biomart human-mouse homologues: List of genes with identified mouse-human homologs; https://www.ensembl.org/index.html

PsychENCODE data: Developmental gene expression; http://development.psychencode.org/

FreeSurfer fsaverage surfaces: https://surfer.nmr.mgh.harvard.edu/

MNI152 T1w 1mm template from FSL: https://fsl.fmrib.ox.ac.uk/fsl/fslwiki/FSL

Schaefer400 17 network atlas: https://github.com/ThomasYeoLab/CBIG/tree/master/stable_projects/brain_parcellation/Schaefer2018_LocalGlobal

Melbourne subcortical atlas: https://github.com/yetianmed/subcortex

Processed and collated data will be available at https://doi.org/10.5281/zenodo.8285838

Human research participants

Policy information about studies involving human research participants and Sex and Gender in Research.

Reporting on sex and gender	HCP data collected information on biological sex. Our interest was population level patterns in thalamic organisation, so we did not consider differences by sex.
Population characteristics	The primary dataset comprised 1080 healthy young adults ranging between 22 and 37 years of age (585 females). The HCP aimed to recruit a cohort that was generally representative of the population at large, thereby capturing a wide range of variability in healthy individuals with respect to behavioral, ethnic, and socioeconomic diversity. See Van Essen et al., 2013 (Neuroimage) for further details. We selected a subset of 100 unrelated participants, of which 76 passed quality control (46 females, age mean ± SD: 28.39 ± 3.95).
Recruitment	Details regarding the recruitment process of the HCP dataset can be found in Van Essen et al., 2013 (Neuroimage).

Ethics oversight	The HCP dataset subject recruitment procedures and informed consent forms, including consent to share de-identified data, were approved by the Washington University institutional review board. Full details can be found in Glasser et al., 2013 (Neuroimage) and Van Essen et al., 2013 (Neuroimage).

Note that full information on the approval of the study protocol must also be provided in the manuscript.

Field-specific reporting

Please select the one below that is the best fit for your research. If you are not sure, read the appropriate sections before making your selection.

☒ Life sciences ☐ Behavioural & social sciences ☐ Ecological, evolutionary & environmental sciences

For a reference copy of the document with all sections, see nature.com/documents/nr-reporting-summary-flat.pdf

Life sciences study design

All studies must disclose on these points even when the disclosure is negative.

Sample size	Due to computational restrictions, we selected 100 unrelated subjects from all 972 subjects who had diffusion data available for the HCP data.
Data exclusions	We excluded participants if they had an inconsistent spatial distribution of seeds to other participants. This was done by assessing for each of the initial seeds, if they were inside or outside the participants own thalamic mask (defined using tissue segmentation with FreeSurfer). If this resulting vector (1 if a seed was inside the mask, 0 if outside) was not highly correlated with other participants (mean r<0.7), these were excluded (n = 24). 76 participants remained for further analysis.
Replication	We replicated the result in the mouse using data from the Allen mouse brain project. We also compared the PCA gene loadings to those obtained by Philllips et al., 2019 (Nature Neuroscience) and found a strong correlation, which also demonstrated our findings replicate.
Randomization	No experimental conditions requiring randomization were applied in the study.
Blinding	No blinding was required as no experimental groups were used in this study

Reporting for specific materials, systems and methods

We require information from authors about some types of materials, experimental systems and methods used in many studies. Here, indicate whether each material, system or method listed is relevant to your study. If you are not sure if a list item applies to your research, read the appropriate section before selecting a response.

Materials & experimental systems

n/a	Involved in the study	
☒	☐	Antibodies
☒	☐	Eukaryotic cell lines
☒	☐	Palaeontology and archaeology
☒	☐	Animals and other organisms
☒	☐	Clinical data
☒	☐	Dual use research of concern

Methods

n/a	Involved in the study	
☒	☐	ChIP-seq
☒	☐	Flow cytometry
☐	☒	MRI-based neuroimaging

Magnetic resonance imaging

Experimental design

Design type	Diffusion
Design specifications	Diffusion and structural scans were collected at rest.
Behavioral performance measures	N/A

Acquisition

Imaging type(s)	Diffusion, structural
Field strength	Human MRI: 3T
Sequence & imaging parameters	HCP data were acquired on a customized Siemens 3T "Connectome Skyra" scanner at Washington University in St Louis, Missouri, USA using a multi-shell protocol for the DWI: 1.25mm3 isotropic voxels, repetition time (TR) = 5520 ms, echo time (TE) = 89.5 ms, field-of-view (FOV) of 210 × 180 mm, 270 directions with b = 1000, 2000, 3000 s/mm2 (90 per b value), and 18 b = 0 volumes. Structural T1-weighted data were collected using 0.7mm3 isotropic voxels, TR = 2400 ms, TE = 2.14 ms, FOV of 224 × 224 mm.
Area of acquisition	Whole-brain scan

Diffusion MRI ☒ Used ☐ Not used

Parameters: Human DW-MRI: The spatial resolution was 1.25 mm isotropic, TR was 5500 ms, TE was 89.50 ms, the b-values were 1000, 2000, and 3000 s/mm2, and the total number of diffusion sampling directions was 90, 90, and 90 for each of the shells, in addition to 6 b0 images

Preprocessing

Preprocessing software	FreeSurfer (v5.3.0); FSL (5.0.11); MRtrix3 (3.0.15); Python (v3.8.5), nibabel (v3.0), parcellation_fragmenter (https://github.com/miykael/parcellation_fragmenter)
Normalization	HCP: normalization of mean b0 image across diffusion acquisitions
Normalization template	Thalamic seeds were initially defined in MNI152 space before being warped to individual subject space.<br><br>fsaverage6 was used to represent all neuromaps data on the cortical surface
Noise and artifact removal	HCP: correction for EPI susceptibility and signal outliers, eddy-current-induced distortions, slice dropouts, gradient nonlinearities and subject motion
Volume censoring	None

Statistical modeling & inference

Model type and settings	For the human data, a PCA was employed on the concatenated thalamocortical connectivity (as derived from tractography data) and thalamic gene-expression matrices. For the mouse, the PCA run on the concatenated thalamocortical connectivity (as derived from axonal tracing data) and thalamic gene-expression matrices
Effect(s) tested	The PCA loadings for the human data were correlated against 72 cortical maps (obtained from neuromaps, significance determined via spin-tests).<br>The PCA loadings for the mouse data were correlated against 9 cortical maps (obtained from an open access dataset).<br>Each genes pattern of expression was correlated with PC1, PC2, and PC3 scores (significance determined via spin-tests). The top 100 positively and negatively correlated genes were used for subsequent enrichment analysis.<br>Enrichment analysis was used to examine how the top 100 positive/negative genes were associated with a) cellular classes, b) disease, and c) development.

Specify type of analysis: ☐ Whole brain ☐ ROI-based ☒ Both

Anatomical location(s): Thalamus: a thalamic mask was defined in MNI152 space using the segmentation by Tian et al., 2020 (Nature Neuroscience). A grid of coordinates 1.75mm apart in this mask was then defined. Seeds that had a consistent spatial location (located within individual thalamic masks for 85% of participants) were retained (n = 921).

Cortex: a random parcellation of 250 regions was defined in the left hemisphere. This parcellation ensured that each region had approximately the same surface area and was calculated using the parcellation_fragmenter Python toolbox. The parcellation was defined on the fsaverage surface and spherically registered to each participants cortical surface.

Statistic type for inference (See Eklund et al. 2016)	Correction for spatial autocorrelations in the human data was performed using spin-tests for both the thalamic (to establish which genes were significantly associated with the PC1 thalamic scores; 1000 permutations) and cortical (to establish which cortical maps were significantly correlated with the PC1 cortical loadings; 10000 permutations) data.
Correction	FDR was computed for all gene enrichment results.

Models & analysis

n/a	Involved in the study
[x]	[ ] Functional and/or effective connectivity
[x]	[ ] Graph analysis
[ ]	[x] Multivariate modeling or predictive analysis

Multivariate modeling and predictive analysis	PCA was performed of the concatenated thalamocortical connectivity and thalamic gene-expression matrices. The scores and loading were used to investigate gradients of gene-expression/thalamocortical connectivity across the thalamus and cortex in both the mouse and human data.<br><br>Differential expression was determined through pairwise comparison over nine developmental windows to determine genes that where enriched either prenatally or postnatally (a minimum of three significant pairwise differences were needed for a gene to be considered enriched for at least one of these timepoints). We used generalised additive models to do so. Briefly, genes expression was modelled as a nonlinear function of age with sex and RNA integrity number acting as fixed effects, along with a random intercept to account for sample-specific variation. The nonlinear function was specified to use a natural cubic spline with four knots evenly spaces across the age span for smoothness. AIC and BIC were used to evaluate model performance. Age-corrected relative gene-expressed was then calculated using the residuals of the best-fit nonlinear mixed model.
